# Harmful effects of mechanical ventilation on neurocognitive functions

**DOI:** 10.1186/s13054-019-2546-y

**Published:** 2019-08-06

**Authors:** Federico Bilotta, Giovanni Giordano, Paola Giuseppina Sergi, Francesco Pugliese

**Affiliations:** Department of Anesthesiology, Critical Care and Pain Medicine, Sapienza University of Rome, Rome, Italy

Whether mechanical ventilation (MV) induces neurotoxicity that can trigger or accelerate chronic cognitive disorders is controversial [1, 2]. The relationship between MV and neurocognitive impairment—that persisted at hospital discharge and at 1-year follow up—was first reported in 1999 in MV-treated ARDS patients [3]. Since then, several preclinical and clinical studies have investigated the mechanisms, localization, and timing of brain damage induced by MV and possible preventive/therapeutic strategies.

## MV-induced brain damage: mechanisms, localization, and timing

Alveolar stretching, PaO_2_/PaCO_2_ abnormalities, and cytokine and chemokine expression in the lungs are the pivotal mechanisms that possibly contribute to MV-induced brain damage [4–9] (Table 1). Alveolar stretching promotes the production of systemic and cerebral mediators (cytokines, chemokines, reactive oxygen species, microglia, and immune system activation) that lead to neuroinflammation, dopaminergic neuronal signaling, and beta amyloid synthesis [4–9]. Relevance of MV-induced alveolar stretching in brain damage is witnessed by production of cerebral beta-amyloid and Alzheimer’s disease-like brain degeneration (cerebral amyloid β peptide accumulation, neuroinflammation, and blood-brain barrier dysfunction) even after relatively “short-term MV” (after 4 h MV in mice)—as reported in a recent study published in *Critical Care* [8]. The PaO_2_/PaCO_2_ abnormalities—including hypo and hyper O_2_ and/or CO_2_ that often represent the indication to establish MV and can persist during MV—might contribute to promote neuronal death, cerebral oxidative stress, and changes in brain blood flow that worsen brain damage [10, 11]. Increased systemic inflammatory mediators produced by the lungs during MV (“biotrauma”) have been detected in several preclinical studies and include TNF alpha, IL6, IL10, IL1 beta, MCP1, and MIP2, which have a proven neuroinflammatory role [4, 5, 7]. Furthermore, MV directly triggers brain inflammation inducing cerebral expression of proinflammatory cytokines (TNF-alpha, IL-1beta, and IL-6) and microglia activation [6, 7, 11]. Of note, the increase in some cerebral inflammatory mediators (TNF-alpha) is proven to be higher when larger tidal volume is applied [5]. Also, the immune system modulation has a key role in MV-induced neuroinflammation, and in preclinical models, an increase in Toll-like receptor 4 (TLR-4) expression (a component of the innate immune system with a role in neuronal survival, neuroplasticity, memory, learning, and cognitive decline) has been detected in the hippocampus after 6 h of MV [7]. Of note, the increase in brain TLR4 expression, and the related increase in plasma and hippocampus cytokines (IL1beta, IL6, TNF alpha) concentration, predicts the extent of memory impairment [7]. The lung-brain vagal reflex induced by MV can also contribute activating dopamine receptors type 2 (DRD2) in the hippocampus and leading to neuronal apoptosis [9].

**Table 1:** Mechanisms of MV-induced brain damage

Alveolar stretching	Systemic and cerebral mediators of neuroinflammation	Systemic release of lung-produced inflammatory mediators (“biotrauma”)
		Local activation of brain inflammatory mediators
		Immunomediated: Toll-like receptor expression
	Afferent neuronal signal: DRD2	
	Beta amyloid production	
PaO_2_/PaCO_2_ abnormalities	Hypo/hyperoxia	
	Hypo/hypercapnia	

Alveolar stretching—through mechanotransduction—induces neuroinflammation with the release of mediators

Some evidence brought conflicting results on MV-induced neurocognitive decline. While alveolar stretch induced by MV promotes de novo synthesis of adhesion molecules in the lung, liver, and kidneys, there is no evidence of such effect in the brain [12]. Furthermore, several of the studies that tested the impact of MV on the brain and neurocognitive functions have limitations that make recorded results not immediately suitable for the clinical realm and lack to discriminate for possibly confounding factors. Last but not the least, the histo-pathological damage, especially when it occurs in the brain, does not necessarily translate into a clinically detectable impairment of neurocognitive functions. For example, a study from Kamuf and coll showed no relationship between MV and an increase in inflammatory mediators eventually associated with brain injury [6].

Clinical relevance of PaO_2_/PaCO_2_ abnormalities and their impact on cognitive function in MV-treated patients has been reported by several studies [10, 13]. Hypoxia—frequent in patients in whom MV is indicated—has been hypothesized to be the most relevant cause of long-term cognitive impairment in patients with ARDS, and it is associated with increased mortality in patients with acute brain damage that require MV [3, 13]. It affects neuronal oxidative phosphorylation, and some brain areas—as the hippocampus—result to be more vulnerable to hypoxic damage and are keener to develop atrophy after hypoxic events [13]. Hypercapnia—another possible indication for MV—causes cerebral vasodilatation with consequent increase of intracranial pressure, and it is associated with mild cognitive impairment in MV preclinical and clinical models [3, 13]. On the other hand, also hyperoxia—which sometime occurs during MV—is a possible mechanism of brain damage since it impairs cerebral autoregulation, reduces CMRO_2_, and is associated with delayed visual evoked potentials [3, 13]. Hypocapnia—a possible iatrogenic complication of MV—induces cerebral vasoconstriction, reduces cerebral compliance, and is associated with a poorer neurological outcome in MV experimental models [10, 13].

## Monitoring and prevention of MV-induced neurocognitive functions decline

Several strategies have been tested to prevent MV-induced brain damage and the related neurocognitive decline; these include neuroinflammatory scavengers, immune modulation, to blunt the dopaminergic neuronal signaling with DRD2-blockers, and lung protective ventilation strategies. The use of scavenger receptor class A has been extensively tested in Alzheimer disease and might play a role in the prevention of MV-induced neurocognitive functions decline. Immune modulation with TLR-4 antagonist has been proven in preclinical models to effectively reduce MV-related neuroinflammation and the extent of cognitive disorders [7]. The use of selective DRD2-blocker (haloperidol) has been successfully tested in experimental animals and is proven to restore the activity of cell survival pathways in the hippocampus, potentially reducing neuronal apoptosis [9]. Protective lung ventilation, with the use of low tidal volume in patients hospitalized after resuscitation for cardiac arrest, associates with better neurocognitive outcome [14]. Optimal PaO_2_/PaCO_2_ management correlates with better neurocognitive status in patients with acute brain injury treated with MV [10, 13].

## Conclusions

Growing preclinical and clinical evidence suggest that MV induces harmful effects on neurocognitive functions. It is not easy to discriminate whether these changes should be interpreted as direct MV-induced damage or as result of perioperative/critical care environment and preexisting frailty. Given the importance to preserve patient’s cognitive functions, we consider it necessary to define a work-up dedicated to monitor neuroinflammation during MV that might include neuroinflammatory scavengers, immune modulation, DRD2-blockers, and lung “neuroprotective” MV management. The latter should encompass optimal MV setting, including low tidal volume, the lowest FiO_2_ needed, to maintain—when possible—PaCO_2_ within normal values and to warrant the shortest MV exposure.

## Data Availability

Not applicable.

## Abbreviations

ARDS: Acute respiratory distress syndrome
DRD2: Dopamine receptor D2
FiO_2_: Fraction of inspired oxygen
ICU: Intensive care unit
IL-10: Interleukin 1
IL-1beta: Interleukin 1-beta
IL-6: Interleukin 6
MCP-1: Monocyte chemoattractant protein
MIP-2: Macrophage inflammatory protein
MV: Mechanical ventilation
PaCO_2_: Arterial partial pressure of carbon dioxide
PaO_2_: Arterial partial pressure of oxygen
TLR4: Toll-like receptor 4
TNF-alpha: Tumor necrosis factor-alpha
